# Pharmacological Basis for the Medicinal Use of *Lepidium sativum* in Airways Disorders

**DOI:** 10.1155/2012/596524

**Published:** 2012-01-15

**Authors:** Najeeb-ur Rehman, Arif-ullah Khan, Khalid M. Alkharfy, Anwarul-Hassan Gilani

**Affiliations:** ^1^Natural Product Research Unit, Department of Biological and Biomedical Sciences, The Aga Khan University Medical College, Karachi 74800, Pakistan; ^2^Department of Pharmacology, Faculty of Pharmacy, University of Karachi, Karachi 75270, Pakistan; ^3^Institute of Pharmaceutical Sciences, Kohat University of Science and Technology, Kohat 26000, Pakistan; ^4^Department of Clinical Pharmacy, College of Pharmacy, King Saud University, Riyadh 11451, Saudi Arabia

## Abstract

*Lepidium sativum* is widely used in folk medicine for treatment of hyperactive airways disorders, such as asthma, bronchitis and cough. The crude extract of *Lepidium sativum* (Ls.Cr) inhibited carbachol (CCh, 1 *μ*M-) and K^+^ (80 mM-) induced contractions in a pattern similar to that of dicyclomine. Ls.Cr at 0.03 mg/mL produced a rightward parallel shift of CCh curves, followed by nonparallel shift at higher concentration (0.1 mg/mL), suppressing maximum response, similar to that caused by dicyclomine. Pretreatment of tissues with Ls.Cr (0.1–0.3 mg/mL) shifted Ca^++^ concentration-response curves (CRCs) to right, as produced by verapamil. Ls.Cr at low concentrations (0.03–0.1 mg/mL) caused leftward shift of isoprenaline-induced inhibitory CRCs, like that caused by rolipram, a phosphodiesterase (PDE) inhibitor. These results indicate that bronchodilatory effect of *Lepidium sativum* is mediated through a combination of anticholinergic, Ca^++^ antagonist and PDE inhibitory pathways, which provides sound mechanistic background for its medicinal use in the overactive airways disorders.

## 1. Introduction

Most of the disorders of respiratory system results from the hyperactivity of airways. Asthma is a major congestive respiratory disorder, characterized by episodic wheezing, cough, and chest tightness, associated with airflow obstruction. The worldwide prevalence of asthma has been increasing particularly in children. According to the World Health Organization, it affects about 5–10% of adults and 10% of children globally [1]. Cough is a spasmodic contraction of the thoracic cavity that results in abrupt release of air from the lungs. Excessive cough is one of the most common symptoms for which the patients seek medical care and may represent up to one-third of a pulmonologist's outpatient practice referrals [2].

The conventional therapy for these congestive airways disorders particularly asthma is not always safe, efficacious and is beyond the access and/or affordability of large proportion of world population, who look for alternative therapeutic measures. Phytotherapy is the most popular alternative remedy, and a number of traditional systems of medicine are heavily based on the use of herbs as medicine [3, 4]. At the same time, there is a global revival of interest in the use of botanicals, and the physicians of the modern medicine are now beginning to accept the traditional remedies once they are scientifically validated [5, 6]. 

*Lepidium sativum* Linn. belongs to family Cruciferae (cabbage family) and is commonly known as “Common cress,” “Garden cress,” or “Halim.” The plant is called “Hab el Rashaad” or “Thufa” in Saudi Arabia and is a popular herbal plant grown in many regions of Saudi Arabia, such as Hijaz, AL-Qaseem, and the Eastern Province [7, 8]. In Europe and America, the leaves are used in salad. In various countries of Africa, *Lepidium sativum* seeds are thought to be an effective medicinal remedy to cure respiratory disorders, like bronchitis and asthma [9]. The plant is cultivated as culinary vegetable all over Asia [10]. In South Asia, it is used in traditional medicine to treat asthma, bronchitis, and cough and is considered useful as abortifacient, antibacterial, aphrodisiac, diuretic, expectorant, gastrointestinal stimulant, gastroprotective, laxative, and stomachic [11, 12].

The plant is known to contain imidazole, lepidine, semilepidinoside A and B [13], *β*-carotenes, ascorbic acid, linoleic acid, oleic acid, palmitic acid, stearic acid [14], sinapic acid and sinapin [15]. *Lepidium sativum *is reported to exhibit antihypertensive [16], diuretic [17], anti-inflammatory, analgesic, anticoagulant [18], antirheumatic [19], hypoglycemic [20], laxative, prokinetic [21], antidiarrheal, and antispasmodic [22] properties. It has been shown to possess antiasthmatic [23] and bronchodilatory [24] potential in preliminary studies, but there is no report available in the literature on the pharmacological basis for its medicinal use in airways disorders. In this study, we explored the possible mechanisms underlying its airways relaxant effect, mediated through multiple pathways, including inhibitory effect on muscarinic receptors, Ca^++^ channels and phosphodiesterase (PDE) enzyme; thus, this study provides sound pharmacological rational for its effectiveness in the airways hyperactive disorders.

## 2. Materials and Methods

### 2.1. Plant Material and Extraction

Seeds of *Lepidium sativum* were purchased from herbal store (Bin Menqash, Riyadh, Saudi Arabia) in March, 2010. The plant was authenticated by Dr. Mohammed Yusuf, King Saud University, and the specimen has been preserved at the herbarium of the College of Pharmacy, King Saud University, Riyadh, Saudi Arabia and also at Natural Product Research Unit, Department of Biological and Biomedical Sciences, Aga Khan University, Karachi with voucher no. Ls-SE-04-10-98. The *Lepidium sativum* seeds were soaked in 70% methanol for three days [25] and then filtered through muslin cloth and Whatman filter paper (Maidstone, UK). This procedure was repeated three times, and all the filtrates were pooled and evaporated on rotary evaporator (model RE-111, Buchi, Flawil, Switzerland) under reduced pressure (−760 mm Hg) to obtain the crude extract of *Lepidium sativum *(Ls.Cr), yielding approximatly 15%.

### 2.2. Chemicals and Animals

Atropine sulphate, carbachol (CCh), dicyclomine, isoprenaline, verapamil, and rolipram were purchased from Sigma Chemicals Company, St. Louis, MO, USA. Guinea-pigs (500–550 g) of either sex and local breed were kept at the Animal House of the Aga Khan University, maintained at 23–25°C, and were given standard diet and tap water. Experiments were performed in compliance with the rulings of the Institute of Laboratory Animal Resources, Commission on Life Sciences, National Research Council [26] and approved by Ethical Committee of the Aga Khan University.

### 2.3. Guinea-Pig Trachea

Tracheal ring strips obtained from guinea-pigs sacrificed by cervical dislocation were mounted individually in 20 mL tissue bath containing Kreb's solution, at 37°C and aerated with carbogen. A tension of 1 g was applied to each of the tissues and equilibrated for 1 hr before the addition of any drug. Carbachol (1 *μ*M) and K^+^0020(80 mM) were used to induce sustained contractions, and relaxant activity of crude extract was assessed by adding in cumulative fashion. High K^+^ (**>**30 mM) is known to cause smooth muscle contractions through opening of voltage-dependent L-type Ca^++^ channels, thus allowing influx of extracellular Ca^++^ causing a contractile effect and the substance causing inhibition of high K^+^-induced contraction is considered as inhibitor of Ca^++^ influx [27, 28]. The antimuscarinic effect of plant extract was investigated through constructing CCh-concentration-response curves (CRCs) in its presence [29]. The Ca^++^ channel blockade (CCB) effect of test substance was confirmed via building Ca^++^-CRCs in the Ca^++^-free medium [28]. The presence of PDE inhibitory effect was evaluated indirectly through constructing isoprenaline-induced inhibitory CRCs against CCh-induced contractions in absence and presence of plant material, as described previously [30]. Isometric responses were recorded on a Grass model 7 Polygraph (Grass Instrument Company, Quincy, MA, USA).

### 2.4. Statistical Analysis

The median effective concentrations (EC_50_) with 95% confidence intervals (CIs) are provided. CRCs were analyzed by nonlinear regression using GraphPad program (GraphPAD, San Diego, CA, USA).

## 3. Results

### 3.1. Inhibitory Effect of Ls.Cr on CCh and High K^+^-Induced Contractions

**Figure 1:**
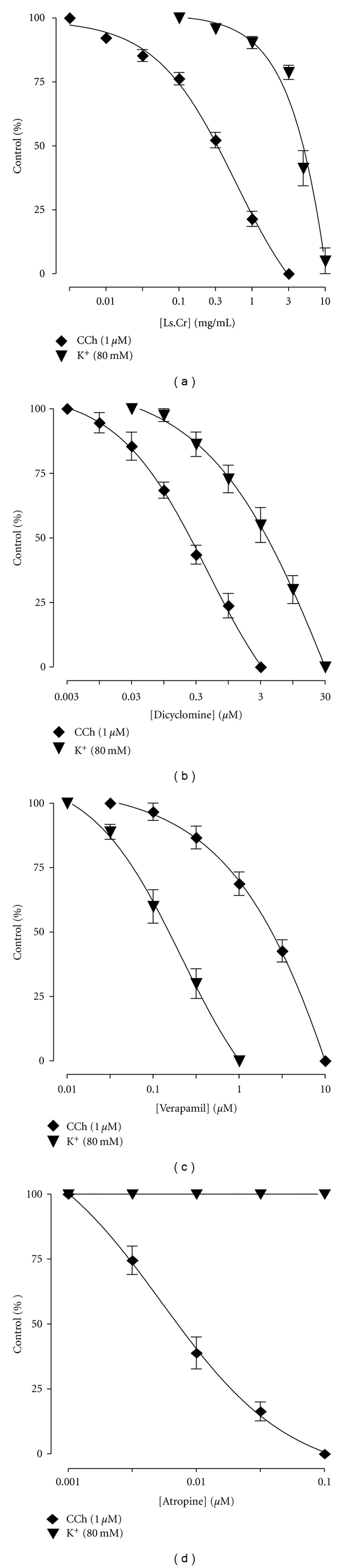
Concentration-response curves showing comparison (a) crude extract of *Lepidium sativum* (Ls.Cr), (b) dicyclomine, (c) verapamil, and (d) atropine for the inhibitory effect against carbachol (CCh) and high K^+^-induced contractions in isolated guinea-pig tracheal preparations, *n* = 3–5.

When tested against CCh (1 *μ*M-) and K^+^ (80 mM-) induced contractions, Ls.Cr inhibited the CCh-induced contractions at lower concentration with EC_50_ value of 0.32 mg/mL (0.30–0.44, 95% CI), compared to its effect against K^+^ (80 mM) with EC_50_ value of 5.4 mg/mL (4.3–7.2) as shown in Figure 1(a). Dicyclomine also showed a similar pattern of inhibitory effect (Figure 1(b)) with respective EC_50_ values of 0.28 *μ*M (0.20–0.40) and 4.6 *μ*M (3.0–7.2), whereas verapamil was more potent against K^+^-induced contractions with EC_50_ value of 0.14 *μ*M (0.10–0.20), when compared with CCh-induced contractions (2.7 *μ*M (1.7–3.5)) as shown in Figure 1(c). Atropine only relaxed the CCh (1 *μ*M-) induced contraction, with EC_50_ value of 0.006 *μ*M (0.002–0.01), without any effect on K^+^ (80 mM-) induced contractions (Figure 1(d)), as expected.

### 3.2. Effect of Ls.Cr on CCh Curves

**Figure 2:**
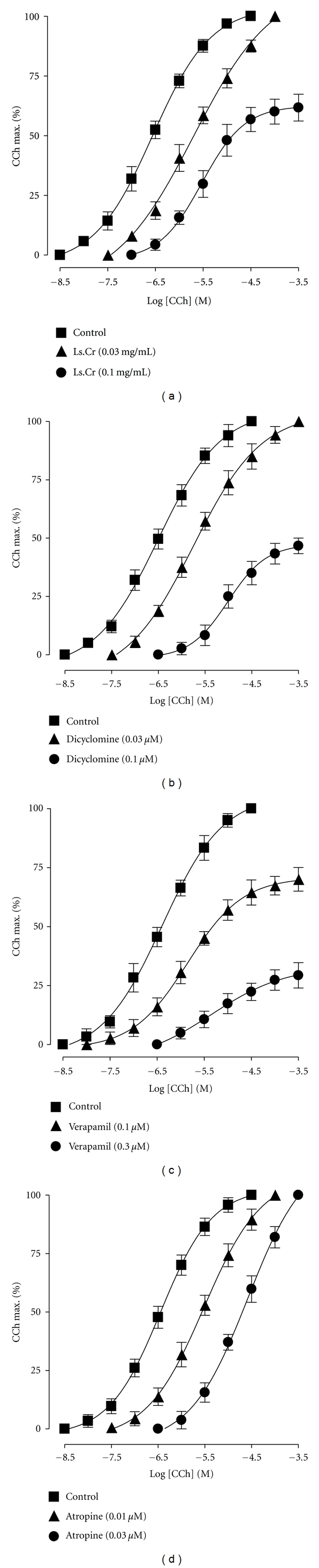
Concentration-response curves of carbachol (CCh) in the absence and presence of different concentrations of (a) crude extract of *Lepidium sativum* (Ls.Cr), (b) dicyclomine, (c) verapamil, and (d) atropine in isolated guinea-pig tracheal preparations, *n* = 3-4.

Pretreatment of the tissues with Ls.Cr at 0.03 mg/mL caused rightward parallel shift of CCh curves, without suppression of maximum contractile response, followed by a non-parallel shift with the suppression of maximum response at next higher concentration, 0.1 mg/mL (Figure 2(a)). Dicyclomine (0.03-0.1 *μ*M) also showed a similar pattern of shift (Figure 2(b)), while verapamil (0.1–0.3 *μ*M) produced a nonparallel rightward shift at both tested concentration with suppression of the maximum response (Figure 2(c)). Atropine (0.01–0.03 *μ*M) caused rightward parallel shift without the suppression of the maximum contractile effect (Figure 2(d)).

### 3.3. Effect of Ls.Cr on Ca^++^ Curves

**Figure 3:**
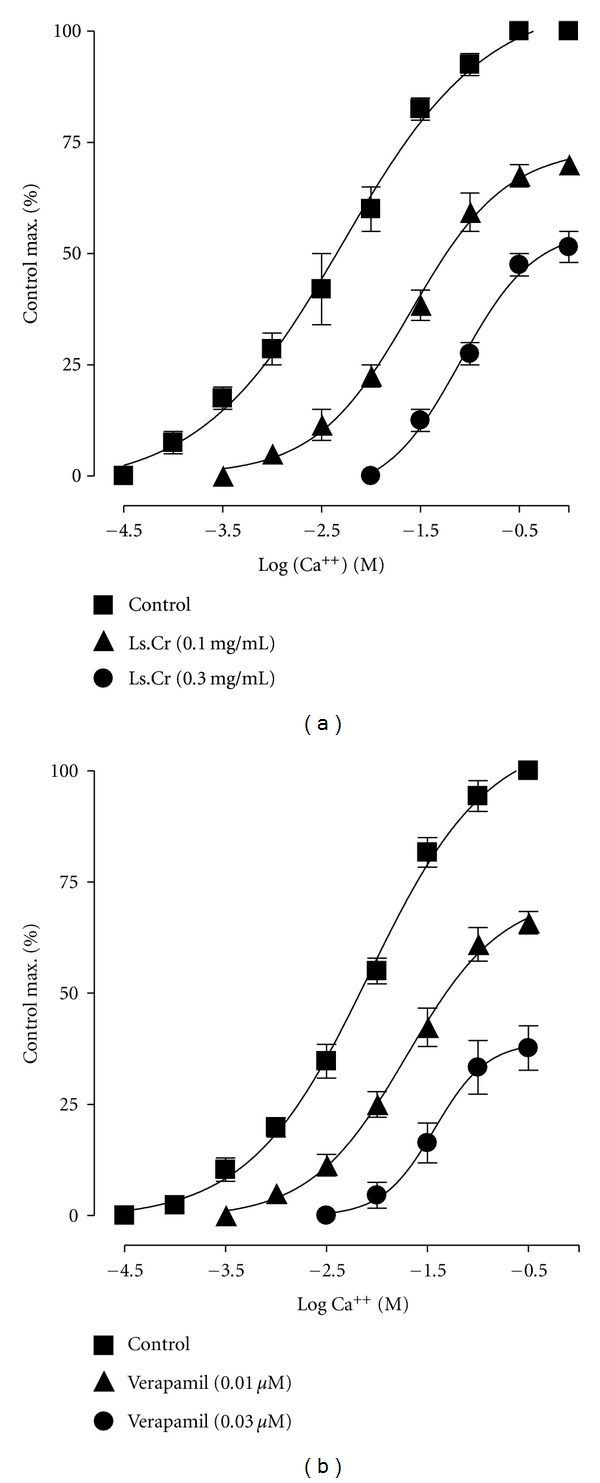
Concentration-response curves of Ca^++^ in the absence and presence of the increasing concentrations of (a) crude extract of *Lepidium sativum* (Ls.Cr) and (b) verapamil in isolated guinea-pig tracheal preparations, *n* = 3-4.

When tested for the possible interaction with Ca^++^ channels, Ls.Cr (0.1–0.3 mg/mL) produced rightward shift in the Ca^++^ curves (Figure 3(a)), similar to that caused by verapamil (Figure 3(b)).

### 3.4. Effect of Ls.Cr on Isoprenaline Curves

**Figure 4:**
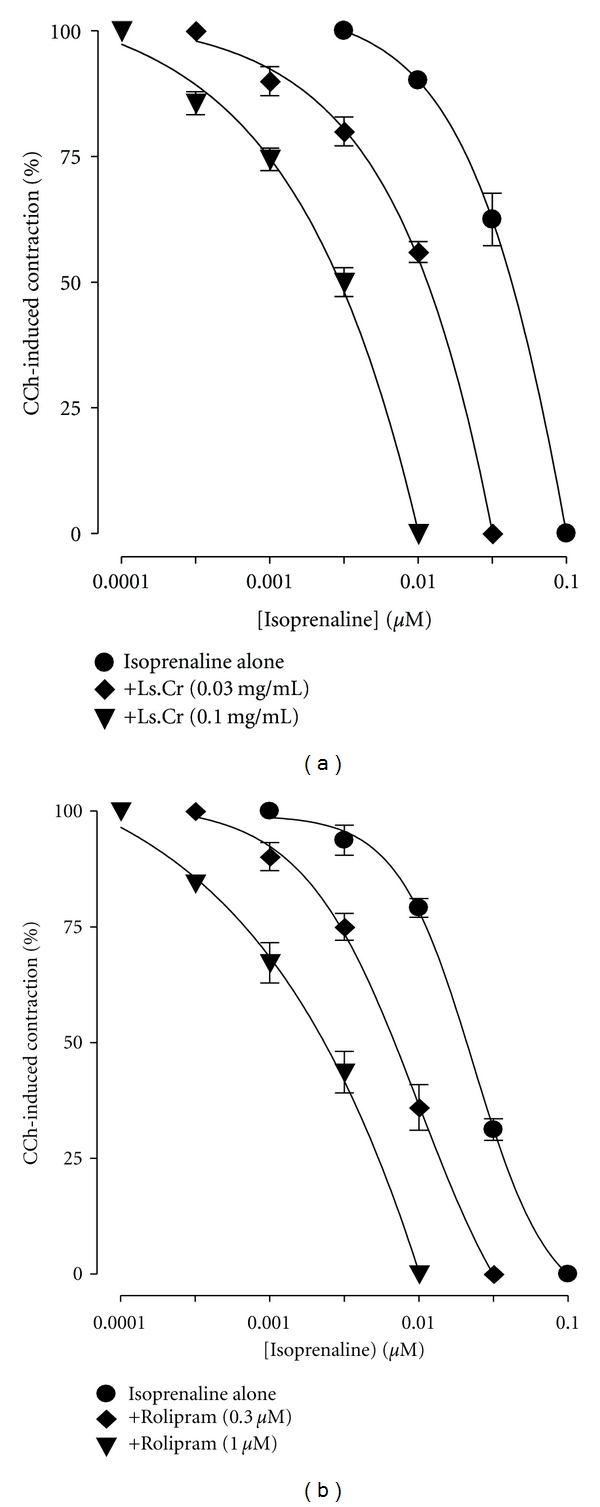
Inhibitory concentration-response curves of isoprenaline against carbachol (CCh)-induced contractions in the absence and presence of different concentrations of (a) crude extract of *Lepidium sativum* (Ls.Cr) and (b) rolipram in isolated guinea-pig tracheal preparations, *n* = 3. The curves obtained by pretreatment of tissues with Ls.Cr and rolipram are significantly different from the respective isoprenaline control curves (*P* < 0.05), Student's *t*-test.

Pretreatment of tissues with Ls.Cr at low concentrations (0.03–0.1 mg/mL) shifted the isoprenaline-induced inhibitory CRCs to the left (Figure 4(a)), showing potentiating effect. Rolipram (0.3–1.0 *μ*M) also caused similar leftward shift of the isoprenaline curves, as shown in Figure 4(b). Dicyclomine, verapamil, and atropine were found devoid of such effect (data not shown).

## 4. Discussion

In view of the medicinal use of *Lepidium sativum* in the hyperactive airways disorders, its aqueous-methanol extract was tested for the possible bronchodilatory effect. In guinea-pig tracheal preparations, plant extract inhibited the CCh- and high K^+^-induced contractions, being more potent against CCh. Dicylomine, a dual blocker of muscarinic receptors and Ca^++^ influx [31], showed a similar pattern of inhibition, while verapamil, a standard Ca^++^ antagonist [32], was more potent against the K^+^-induced contractions than those induced by CCh, while atropine, a muscarinic receptor antagonist [33], relaxed the CCh-induced contractions only. It is thereby suggesting the inhibitory effect on muscarinic receptor and Ca^++^ channels. The presence of anticholinergic and CCB actions was further confirmed, respectively, through constructing the CCh and Ca^++^ CRCs in the presence of different concentrations of the plant extract. A parallel displacement of CCh curves without suppression of the maximum effect was observed at the lower concentration of Ls.Cr, a characteristic of a competitive or specific antagonist, like atropine [34], followed by non-parallel shift with suppression of the maximum effect at higher concentration, pointing towards the presence of nonspecific inhibition, like known with Ca^++^ antagonists [35]. Dicyclomine also shifted the CCh curves to the right, similar to that of the crude extract, while verapamil resulted in rightward but non-parallel shift with suppression of the maximum effect at both the concentrations used. Atropine caused a rightward parallel shift of the CCh curves without suppression of maximum response. Pretreatment of tissues with Ls.Cr shifted the Ca^++^ curves to the right accompanied by the suppression of maximum response, similar to that caused by verapamil, confirming the Ca^++^ antagonistic effect.

 We have experienced that plants with medicinal use in the overactive airways disorders usually possess PDE inhibitory effect, which usually coexists with Ca^++^ antagonist activity [30, 36, 37]. To investigate whether *Lepidium sativum *also exhibits PDE enzyme inhibition component(s), isoprenaline inhibitory CRCs were constructed against CCh-induced contractions by pretreatment of tissues with Ls.Cr, as PDE inhibitors are known to potentiate the isoprenaline effect [38]. The presence of PDE inhibitory effect in *Lepidium sativum *was confirmed when the plant extract potentiated isoprenaline relaxant effect, by causing leftward shift of isoprenaline-induced inhibitory curves, similar to that caused by rolipram, a PDE_4_ inhibitor, predominant enzyme in airways [39]. These findings indicate that bronchodilator effect of *Lepidium sativum *is mediated through combined anticholinergic, CCB, and PDE inhibitory pathways; thus, this study provides sound mechanistic basis for its application in airways hyperactivity disorders. The usefulness of anticholinergics and PDE inhibitors in asthma is well established [40], though major limitation is cardiac stimulation, as a side-effect when applied orally [41, 42]. Interestingly, Ca^++^ antagonists are also useful in bronchoconstriction [43] and are known to exhibit cardiosuppressant effect [44]. The coexistence of CCB constituent(s) with antimuscarinic and PDE inhibitors in *Lepidium sativum *is perhaps meant by nature to offset the tachycardia, usually associated with anticholinergics or PDE inhibitors when used alone. This scenario strengthens the concept that natural remedies possess “effect enhancing and/or side-effects neutralizing” potential in addition to cost-effectiveness and offers merit in evidence-based studies [6, 45]. 

In conclusion, these results show that *Lepidium sativum *offers a unique combination of bronchodilator activities (anticholinergic, Ca^++^ antagonist and PDE inhibitory effects), which may explain its medicinal use in the hyperactive airways disorders, such as cough and asthma. However, further in-depth studies are required to probe nature of chemicals constituents and molecular base of biological activities.
